# Taste-Masked Pellets of Warfarin Sodium: Formulation towards the Dose Personalisation

**DOI:** 10.3390/pharmaceutics16050586

**Published:** 2024-04-26

**Authors:** Lakija Kovalenko, Kirils Kukuls, Marta Berga, Valentyn Mohylyuk

**Affiliations:** Laboratory of Finished Dosage Forms, Faculty of Pharmacy, Riga Stradiņš University, LV-1007 Riga, Latvia

**Keywords:** warfarin, pellets, taste masking, cores, sodium chloride, Kollicoat^®^ Smartseal, salting-out effect

## Abstract

The bitter drug, warfarin, has a narrow therapeutic index (NTI) and is used in paediatrics and geriatrics. The aim of this feasibility study was to formulate the taste-masked warfarin-containing pellets to be applicable for dose personalisation and to improve patient compliance, as well as to investigate the effect of the core type (PharSQ^®^ Spheres M, CELPHERE™ CP-507, and NaCl) on the warfarin release from the Kollicoat^®^ Smartseal taste-masking-coated pellets. The cores were successfully drug-loaded and coated in a fluid-bed coater with a Wurster insert. An increase in particle size and particle size distribution was observed by optical microscopy. In saliva-simulated pH, at the Kollicoat^®^ Smartseal level of 2 mg/cm^2^, none of the pellets demonstrated drug release, confirming their efficient taste-masking. However, in a stomach-simulated pH, a faster drug release was observed from PharSQ^®^ Spheres M- and CELPHERE™ CP-507-coated pellets in comparison with NaCl cores. Additional experiments allowed us to explain the slower drug release from NaCl-containing pellets because of the salting-out effect. Despite the successful taste masking, the drug release from pellets was relatively slow (not more than 91% per 60 min), allowing for further formulation improvements.

## 1. Introduction

The main disadvantage of most oral mass-market drug products is that they do not fit everyone. The reason usually lies in the required personalised approach, including dose personalisation or consumer properties [1,2]. Inappropriate consumer properties are usually associated with unpleasant smells, tastes, swallowing discomfort, or even swallowing problems (such as dysphagia) [3]. Some paediatric age subgroups have contraindications to be treated with tablets and capsules at the same time and, for the geriatric population subgroup, swallowing discomfort is acquired with age because of decreased motor activities [4,5]. It is worth mentioning that swallowing discomfort is observed often, even in healthy adults [6].

The usual real-world approach to solving the swallowing problem is to break or crush the tablet or open the capsule [7]. The problem with this is that the biopharmaceutical properties of drug products can change drastically due to changes in the dissolution profile and the place of drug release [8,9]. In addition, after these unauthorised modifications, the unpleasant smell and taste problems intensify.

One of the drugs that requires both dose personalisation and taste masking [10] is warfarin [11]. Warfarin is a coumarin anticoagulant and a vitamin K antagonist used to treat and originally prevent thromboembolic events and the development of the disease in cases of many different health conditions, such as deep venous thrombosis, cardiomyopathy, and pulmonary embolism. It is also used for thrombosis, stroke, and myocardial infarction prophylaxis, among others. Warfarin inhibits the vitamin K-dependent synthesis of coagulation factors (II, VII, IX, and X) by inhibiting vitamin K reductase, vitamin K epoxide reductase, and anticoagulant proteins C and S, therefore depleting the available vitamin K-dependent activated proteins that play a role in the coagulation process. At the beginning of the treatment, a certain loading dose is needed. Due to the mechanism of action, the first effect is seen gradually, and the full clinical effect can be observed 3–4 days after starting the therapy. Based on the indication and considering its narrow therapeutic index, the general oral dose may vary from 2 to 10 mg once a day, depending on age, weight, genetic polymorphism, and the prothrombin time [12].

Thus, the personalisation of the warfarin dose is demanded. Patients may greatly benefit from changes in dosage using different drug dosage forms as well as oral dosage forms that can still be taste-masked against the bitter taste of warfarin, while keeping the flexibility of personalised dosing [10,11,13].

One of the approaches to solving taste and swallowing problems is to formulate the drug in the form of a liquid suspension or solution. Taste masking is usually achieved by adding taste additives and sweeteners [10]. Despite the relative effectiveness of this method, solutions and suspensions cannot solve the taste-masking problem completely because they cannot prevent the drugs from come into contact with the taste buds [10,14,15].

To the best of our knowledge, the best alternative approach is represented by taste-masked microparticles [14,16,17]. This approach provides better taste masking due to the microparticles, which are insoluble in oral cavity saliva with a pH of 5.8–7.6 [18]. Thus, the drug does not come into contact with the taste buds. The insolubility of particles in oral liquids can be achieved by using polymers with pH-dependent solubility, such as Kollicoat^®^ Smartseal 30D. This polymer is insoluble at the pH of the oral cavity, but dissolves quickly at the pH of the stomach [16,19].

The taste-masked microparticles/pellets can be manufactured by coating drug-containing granules or by drug-layering placebo cores and then applying a taste-masking coating [20,21]. Even though placebo cores are usually perceived as inert, the cores’ properties can affect the drug release from the pellets. For instance, the density of the particles predetermines the number of particles per gram, the size of the cores predetermines the specific surface area, and the solubility and osmolarity can increase the release rate [22,23,24].

While the application of Kollicoat^®^ Smartseal to obtain taste-masking-coated pellets is known, to the best of our knowledge, this polymer has never been investigated before for the preparation of warfarin-containing pellets. Moreover, the effect of the core type on the release of warfarin from the Kollicoat^®^ Smartseal-coated pellets has not yet been investigated.

The aim of this study is to investigate the effect of microcrystalline cellulose, anhydrous dibasic calcium phosphate, and sodium chloride cores of comparable sizes in terms of their effect on the release of warfarin from the Kollicoat^®^ Smartseal taste-masking-coated pellets.

## 2. Materials and Methods

### 2.1. Materials and Reagents

**Figure 1:**
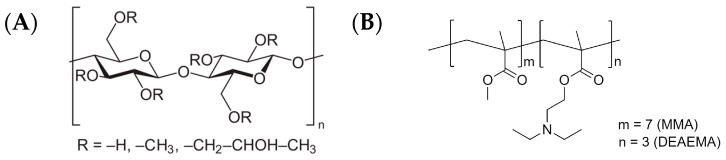
The molecular structure of Methocel™ E5 premium LV (HPMC; (**A**)) and Kollicoat^®^ Smartseal (MMA:DEAEMA; (**B**)).

Warfarin sodium clathrate (batch # WA22003; Alchymars Icm SM Private Ltd., Tamil, Nadu, India) was used as an active pharmaceutical ingredient. Microcrystalline cellulose (MCC) spheroids (CELPHERE™ CP-507 (500–710 µm); Asahi Kasei Co., Tokyo, Japan), dibasic calcium phosphate anhydrous (80 wt.%) and microcrystalline cellulose (20 wt.%) spheroids (PharSQ^®^ Spheres M (500–710 µm); Budenheim KG, Budenheim, Germany) [25,26], and sodium chloride (NaCl; Valdo SIA, Riga, Latvia) sieved particles (500–800 µm fraction) were used as cores of pellets. Hydroxypropyl methylcellulose (HPMC; Methocel™ E5 premium LV; Dow Chemical, Midland, MI, USA) was used as a binder for drug loading (Figure 1A). Co-polymer comprising methyl methacrylate (MMA) and diethylaminoehtylmethacrylate (DEAEMA) in the ratio 7:3 in the form of aqueous dispersion Kollicoat^®^ Smartseal 30D (BASF SE, Ludwigshafen, Germany) was used as a taste-masking coating material (Figure 1B) [27] and dibutyl sebacate (DBS; Merck KGaA, Darmstadt, Germany) as a plasticiser [28]. A synthetic colloidal silicon dioxide (Syloid^®^ 244FP; Grace GmbH, Worms, Germany) was used as anti-tacking/-caking agent. Hydrochloric acid (37%), potassium dihydrogen phosphate, and disodium hydrogen phosphate (analytical grade) were supplied by Merck KGaA (Darmstadt, Germany).

### 2.2. Moisture Content

The moisture content of warfarin sodium clathrate was determined by thermogravimetric analysis (TGA; loss on drying). The accurately weighed sample of approx. 500 mg was exposed to a constant temperature of 180 °C and the weight loss due to the moisture evaporating was recorded (HX204; Mettler Toledo AG, Greifensee, Switzerland). Measurements were done in triplicate, and the moisture and warfarin sodium contents were presented as averages, including the standard deviations (*n* = 3; Av. ± S.D.).

### 2.3. Sieving

NaCl sieved particle fraction (500–800 µm) was obtained by 10 min shaking at an amplitude of 0.5 mm with 3 s intervals using sieves with 500 and 800 µm mesh sizes in a sieve shaker (AS 200; Retsch GmbH, Haan, Germany).

### 2.4. Particle Size Distribution: Laser Diffraction

The particle size distribution as well as the D10%, D50%, and D90% were determined by a laser diffraction particle size analyser using an Aero S module for dry dispersions (Mastersizer 3000, Malvern Instruments, Malvern, UK) at the specified settings: feed rate of 30–80%; hopper gap of 1.0–1.5 mm; and air pressure of 2.0 bar. Approximately 10–15 g of the sample was used for each repetition (*n* = 3).

### 2.5. Microscopic Analysis

The particle size of NaCl, CELPHERE™ CP-507, and PharSQ^®^ Spheres M before/after drug loading and after taste-masking coating was determined with an optical microscope (equipped with lens #4) coupled with a digital camera (BA410E; Motic, Xiamen, China). For each determination, 150 randomly collected particles were measured (Motic Images Plus 3.0 ML; Motic, Xiamen, China). For the almost-cubic NaCl crystals, two sides of the visible square surfaces of each particle were measured and averaged, while for the spheroid particles, the minimum and maximum apparent diameters were measured and averaged. Using the averaged values, the apparent volume and surface area of each particle were calculated, as well as the total surface area and total volume of 150 particles. The mass of 150 particles was determined by weighing 150 randomly collected particles (*n* = 3; not the same particles as mentioned above). To determine the apparent density of particles, the calculated total volume of 150 single particles was divided by the mass of 150 particles. The specific surface area of particles (cm^2^/g) was calculated by dividing the total calculated surface area of 150 single particles by the mass of 150 particles [29].

*D*_10%_, *D*_50%_, and *D*_90%_ sizes were determined from the raw data of microscopic measurements after being graphically represented for all three types of pellets using the span of the size distributions using the following equation [23]:

$$Span=\frac{D_{90\%}-D_{10\%}}{D_{50\%}}$$

### 2.6. Drug Layering

The HPMC containing [13] aqueous-based warfarin sodium clathrate layering solution [30] with a solid content of 30 wt.% (Table 1) was sprayed onto cores in a fluidised bed coater (Mini-Glatt, Glatt GmbH, Binzen, Germany) with a Wurster tube (positioned at 12 mm above the bottom of the processing chamber) to achieve 30 wt.% weight gain (28.7 wt.% weight gain due to the warfarin sodium) per 100 g of cores. The drug loading weight gain (*WG*%) was calculated by the following equation:

$$WG\%=\frac{m_{drug\;layered}-m_{initial}}{m_{initial}}\times 100\%$$

The warfarin sodium layering solution was sprayed at the rate of 1.1–1.7 g/min through a 0.5 mm nozzle at an atomisation air pressure of 1.7 MPa onto cores with a bulk volume of 50 mL which corresponds to 80, 132.3, and 109.1 g of CELPHERE™ CP-507, PharSQ^®^ Spheres M, and NaCl cores, respectively. While the inlet air flow was set at 17.9–18.2 m^3^/h, the inlet air temperature and the pellets temperature were kept at 45 °C and 25–35 °C, respectively (Appendix A, Appendix B and Appendix C) [16,31].

### 2.7. Pellets Coating for Taste Masking

The specific surface area of drug-loaded cores was determined as described above. To achieve the weight gain of 2 mg/cm^2^, drug-layered pellets were coated with taste-masking coating: Kollicoat^®^ Smartseal 30D (30 wt.% of solid content) aqueous dispersion plasticised by DBS (in the concentration of 13% *w*/*w*, based on the polymer; Table 2) [27]. Kollicoat^®^ Smartseal 30D and DBS were mixed for at least 18 h.

The coating process was realised in a fluidised bed coater (Mini-Glatt, Glatt GmbH, Binzen, Germany) with a Wurster tube (positioned at 10 mm above the bottom of the processing chamber). Aqueous latex dispersion of plasticised Kollicoat^®^ Smartseal was sprayed at a rate of 1.3 g/min (for CELPHERE™ CP-507 and NaCl drug-loaded cores) and 2.3 g/min (for PharSQ^®^ Spheres M drug-loaded cores) through the 0.5 mm nozzle at an atomisation air pressure of 1.7 MPa onto cores with a bulk volume of 50 mL, which corresponds to 101.0, 152.8, and 165.5 g of CELPHERE™ CP-507, PharSQ^®^ Spheres M, and NaCl drug-loaded cores, respectively. While the inlet air flow was set at 17.9–18.2 m^3^/h, the inlet air temperature and the pellets temperature were kept at 25 °C and 15–25 °C, respectively (Appendix A, Appendix B and Appendix C). After coating, the pellets were mixed with an anti-tacking/-caking agent, Syloid^®^ 244FP (0.5 wt.%), and cured in an oven at 60 °C overnight [16,28].

### 2.8. Drug Release

Approximately 11.3 mg of CELPHERE™ CP-507-coated pellets, 11.2 mg of PharSQ^®^ Spheres M-coated pellets, and 10.9 or 12.7 mg NaCl-coated pellets (depending on coating WG% to achieve a warfarin dose of 2 mg) were tested in 1000 mL of phosphate buffer solution (PBS, pH 6.8; in accordance with EP) or 0.1 N HCl solution (pH 1.2) at 37 ± 0.5 °C using a USP II paddle apparatus (ATS Xtend™, SOTAX AG, Allschwil, Switzerland) at 100 rpm. The amount of dissolved warfarin was determined spectrophotometrically (Specord^®^ UV-Vis; Analytik Jena AG, Jena, Germany) at a wavelength of 307 nm (C = (Abs. + 0.0045)/0.0432; R^2^ = 0.994) in phosphate buffer and at a wavelength of 283 nm (C = (Abs. − 0.002)/0.0445; R^2^ = 0.997) in HCl solution.

### 2.9. Appearance of Single Pellet upon Exposition in Medium

The appearance of a single pellet in 0.1 N HCl was observed using an optical microscope (lens #4) connected to a digital video camera (BA410E; Motic, Xiamen, China). For better visibility, a black background was used.

### 2.10. Kollicoat^®^ Smartseal Film Testing

A few replicates of 50 µL of Kollicoat^®^ Smartseal 30D were transferred to microscope slides and dried overnight in a vacuum oven at 60 °C. Subsequently, each microscope slide with formed films was immersed in a 0.1 N HCl solution (pH 1.2) with 0, 11.5, and 23% (*w*/*v*) concentrations of NaCl and exposed at static conditions and room temperature. The films were observed periodically.

## 3. Results and Discussion

**Figure 2:**
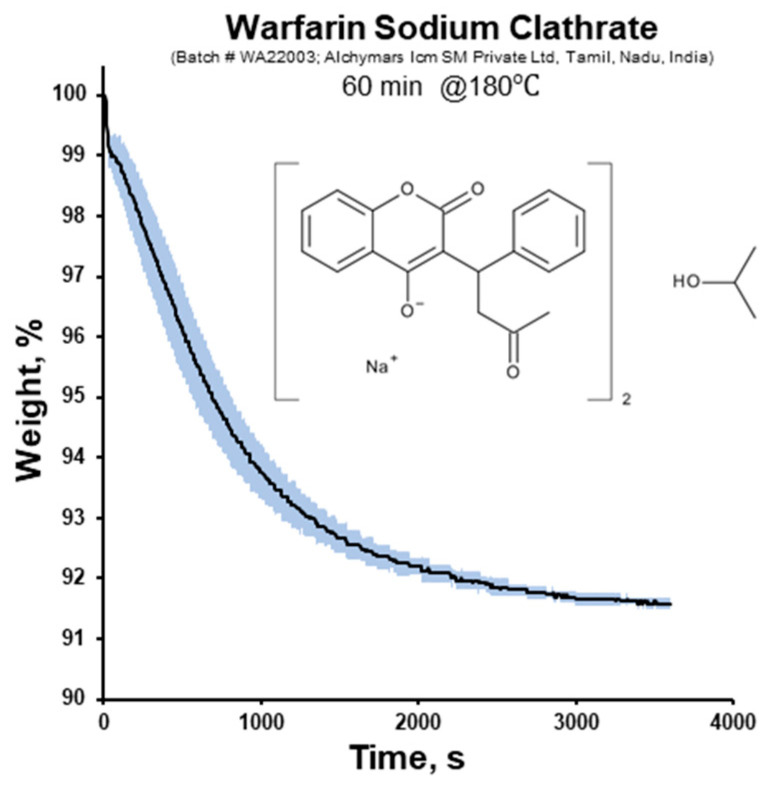
TGA profile of sodium warfarin clathrate (the light colour denotes the S.D.).

In oral dosage forms, the most commonly used form of warfarin is a soluble salt, specifically sodium warfarin, including its sodium warfarin clathrate crystal form. In the sodium warfarin clathrate crystals, the molecule of isopropyl alcohol is trapped in the crystalline structure in a 2:1 molecular proportion, which is equivalent to 92 wt.% of sodium warfarin [32,33,34,35,36]. The experimental results obtained via TGA confirmed this by showing the moisture and warfarin sodium contents at 8.4 ± 0.1 and 91.6 ± 0.1 wt.%, respectively (Figure 2).

In this study, cores with a size that is usually unproblematic upon aqueous polymeric dispersion coating were used in the Wurster fluid-bed coaters [23]. Soluble NaCl cores had a cubic shape, while PharSQ^®^ Spheres M, soluble at certain pHs (such as at pH 6.8 in phosphate buffer solution), and insoluble CELPHERE™ CP-507 cores had a spheroid shape (Figure 2). The cores were successfully drug loaded and coated (Table 1 and Table 2, Appendix A, Appendix B and Appendix C).

**Figure 3:**
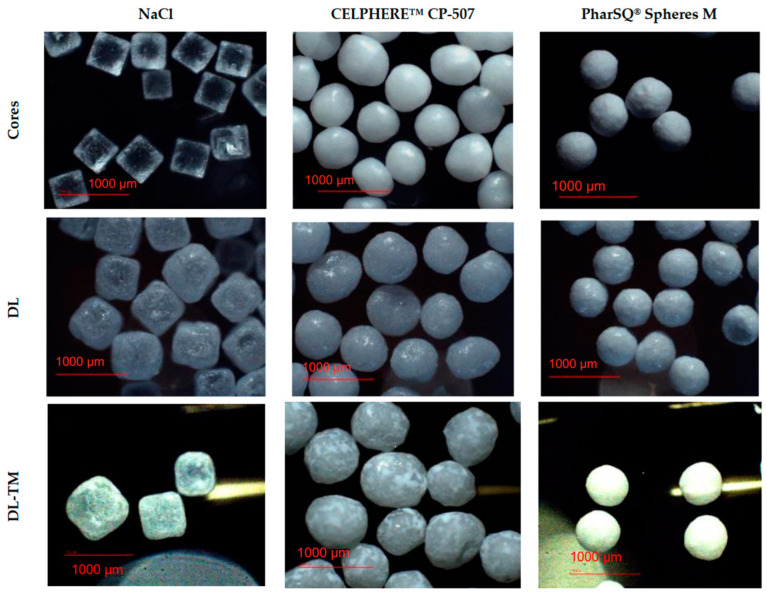
Microscopy of cores, drug-layered and coated pellets.

All pellets were drug-loaded with a 30% warfarin sodium clathrate solution and then coated with the Kollicoat^®^ Smartseal taste-masking coating (Figure 3, Table 3).

**Figure 4:**
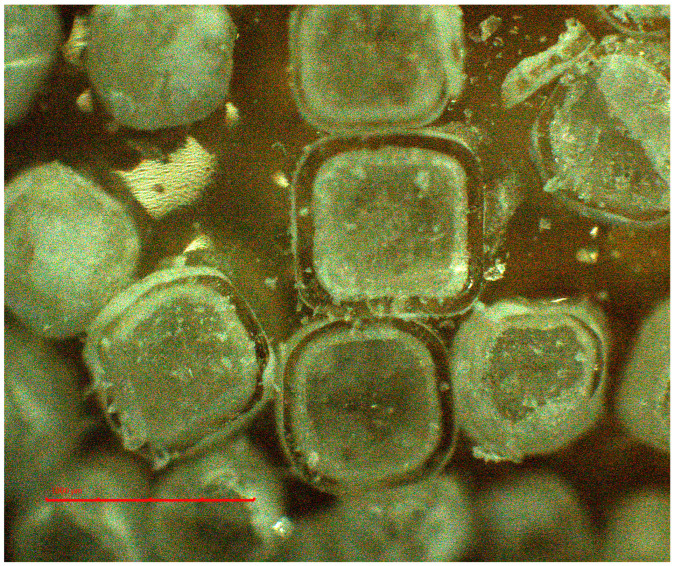
NaCl-containing pellets: the microscopy of pellets’ cross-section.

**Figure 5:**
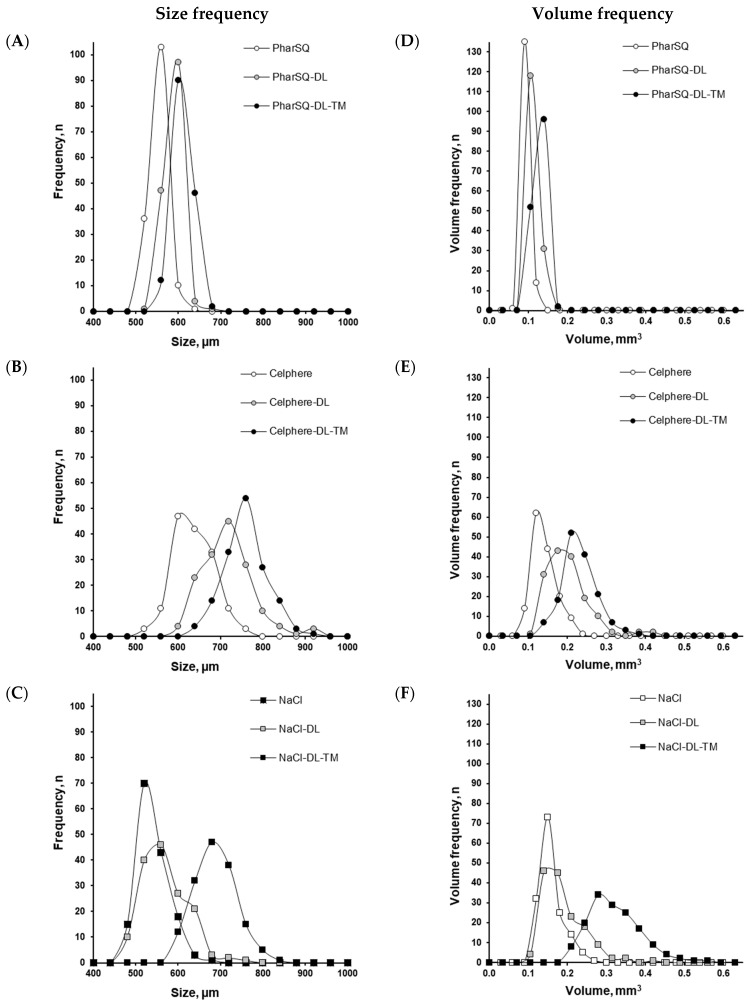
Size ((**A**–**C**) virtual sieve steps equal to 40 µm) and volume ((**D**–**F**) virtual sieve steps equal to 0.035 mm^3^) frequency of pellets with PharSQ^®^ Spheres M (**A**,**D**), CELPHERE™ CP-507 (**B**,**E**), and NaCl ((**C**,**F**), respectively) cores (*n* = 150).

The structure of the pellets, including the drug loading of the cores and coating, is illustrated by the cross-section microscopy of NaCl-containing pellets (Figure 4). Upon drug-loading and coating, the average size of the cores increased from 532 to 570 and to 590 µm for PharSQ^®^ Spheres M, from 615 to 698 and to 738 µm for CELPHERE™ CP-507, and from 521 to 549 and to 668 µm for NaCl (Table 4; Figure 5).

The average thickness of the coating can be assumed based on the difference between the average size of the drug-loaded and taste-masked pellets. Therefore, the assumed average thickness of the taste-masked coating for NaCl, CELPHERE™ CP-507, and PharSQ^®^ Spheres M will comprise 59.5, 20, and 10 µm, respectively.

**Figure 6:**
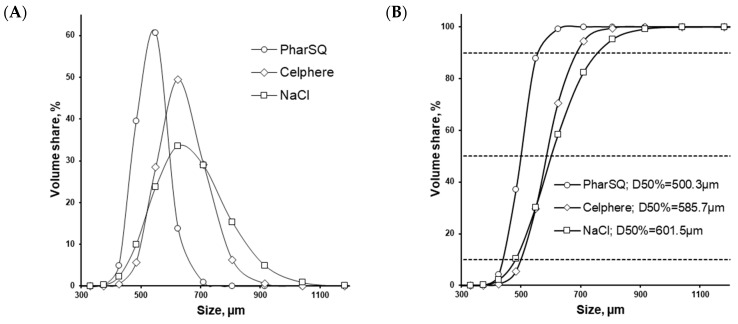
Particle size distribution (**A**) and cumulative volume share (**B**) measured by laser diffraction method.

The particle size (D_50%_) of the initial cores measured with the laser diffraction method agreed with the microscopic method, resulting in 500 µm for PharSQ^®^ Spheres M, 586 µm for CELPHERE™ CP-507, and 602 µm for NaCl (Figure 6, Table 5). The span of the core sizes was the smallest at 0.23 for PharSQ^®^ Spheres M, followed by 0.32 for CELPHERE™ CP-507, with the biggest being 0.46 for the NaCl cores (Table 5). Considering the cubic structure of the NaCl cores (Figure 3), further calculations were done via microscopic observations.

The average volume of the cores increased from 0.079 to 0.097 and to 0.108 mm^3^ for PharSQ^®^ Spheres M, from 0.124 to 0.182 and to 0.213 mm^3^ for CELPHERE™ CP-507, and from 0.143 to 0.170 and to 0.303 mm^3^ for NaCl (Table 4; Figure 5).

The apparent density of the initial NaCl, CELPHERE™ CP-507, and PharSQ^®^ Spheres M cores was different. Due to the sequential drug loading and coating, the PharSQ^®^ Spheres M- and CELPHERE™ CP-507-containing pellets changed their apparent density from 1.60 to 1.69 and 1.88 g/mL, and from 1.24 to 1.26 and 1.33 g/mL, respectively (Table 4). The initial apparent density of the NaCl cores was 2.36 g/mL, while after drug loading and coating, the apparent density (calculated based on the cubic geometry) was 2.25 and 1.39 g/mL, respectively. The drastic decrease in the calculated apparent density of NaCl-containing pellets can be explained by the lower density of the drug and taste-masking layers, as well as by the soft edges and the loss of their cubic-like shape (Figure 3). The shape and apparent density of the cores predetermine the specific surface area of the drug-loaded pellets (Table 4), whereas the calculated specific surface area predetermines the required amount of taste-masking coatings to achieve a desirable coating level.

Practical drug layering and coating (Table 3) resulted in different pellets’ compositions (Table 6). While PharSQ^®^ Spheres M and CELPHERE™ CP-507 cores are well-known in the pharmaceutical industry, NaCl cores are not unusually used. Thus, safety questions could be raised. In the case of NaCl drug-loaded and taste-masked pellets, following calculations, every 1 mg of warfarin sodium will be accompanied by 3.7 mg of NaCl (Table 6) which is equivalent to 1.5 mg of sodium. Considering the daily recommended intake of sodium which is between 2000 and 5000 mg/day [37,38], 1.5 mg comprises 0.08–0.03%, respectively. So, the intake of therapeutic doses of warfarin with NaCl drug-loaded and taste-masked pellets cannot significantly influence the daily intake of sodium and compromise safety.

**Figure 7:**
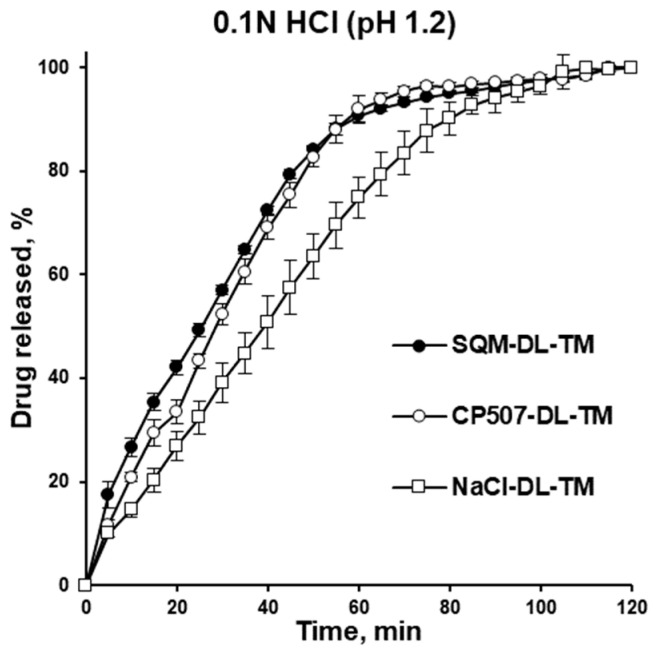
Drug release from drug-loaded and taste-masking-coated pellet in 0.1 N solution of HCl.

Despite the difference in cores and the resulting properties of the pellets (Table 4, Figure 5), none of the pellets demonstrated drug release in the phosphate buffer with a pH of 6.8, which simulates the pH of oral saliva. This confirms the efficiency of the taste masking. The release of warfarin was observed in stomach-simulated conditions (0.1 N HCl solution). The drug release kinetics decreased from PharSQ^®^ Spheres M to CELPHERE™ CP-507 and to NaCl-containing pellets, and the average amount of drug released in 60 min comprised 91, 92, and 75%, respectively (Figure 7). Despite the reported incompatibility of calcium phosphate and warfarin [39], in our study, the complete drug release from the pellets was observed via UV detection. Therefore, the experimental drug release results did not comply with the immediate release requirements for any of the pellets [11].

**Figure 8:**
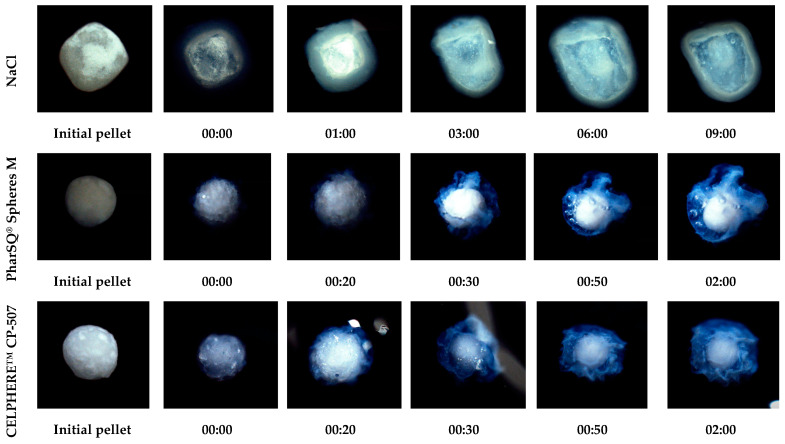
Appearance of drug-loaded and taste-masking-coated pellet upon exposition in 0.05 mL of 0.1 N HCl solution at room temperature. The time represented below the images is in the “minutes:seconds” format.

The behaviour of single pellets upon exposition to a 0.1 N HCl solution without hydrodynamic forces agreed with the drug release from these pellets (Figure 8). At a comparable coating level, the taste-masking-coating release was faster for PharSQ^®^ Spheres M- and CELPHERE™ CP-507- than for NaCl-containing pellets. The drug release kinetic decrease was in full accordance with the increase in the assumed average thickness of the taste-masked coating. Nevertheless, NaCl is known to be osmotically active, and thus faster water absorption and dissolution can be expected due to the osmotically active core.

However, the drug release from the NaCl-coated pellets was the slowest. When the NaCl-coated pellets were put in a 0.1 N HCl solution, the Kollicoat^®^ Smartseal coating turned opalescent instead of clear, and it stayed on the pellets for a much longer time than on the other cores.

**Figure 9:**
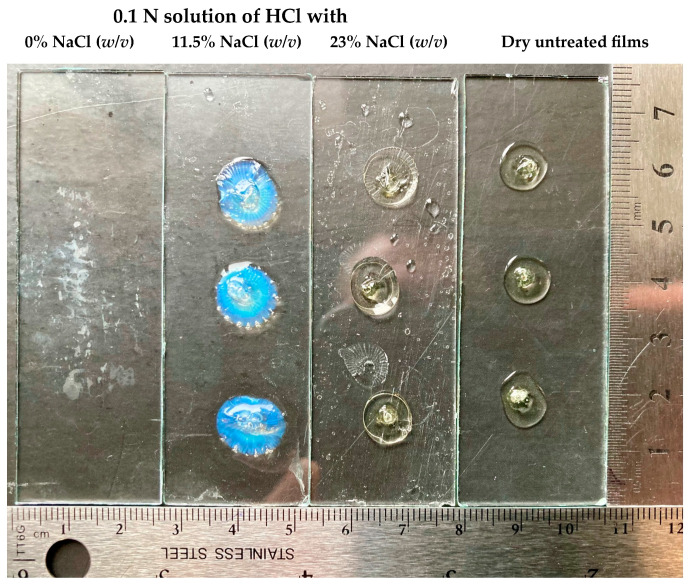
The appearance of Kollicoat^®^ Smartseal films after 4 h and 30 min of exposition in a 0.1 N HCl solution with NaCl concentrations of 0, 11.5, and 23% (*w*/*v*) at room temperature and static conditions.

To reveal the effect of the NaCl core on the drug release, isolated dry Kollicoat^®^ Smartseal films were exposed to a 0.1 N solution of HCl with different concentrations of NaCl at room temperature under static conditions. Interestingly, at a 0% concentration of NaCl, the Kollicoat^®^ Smartseal films dissolved in less than 60 min, while even in 4.5 h, the films remained intact in the solutions with 11 and 23% of NaCl (Figure 9).

The films exposed to an 11.5% concentration of NaCl were swollen and had opalescence, while those exposed to a 23% concentration of NaCl did not swell and were transparent. Being different compared to the other pellets, the NaCl-coated pellets showed similar opalescence upon single-pellet microscopic observation in a 0.1 N solution of HCl (Figure 8). This experiment explained the different behaviours of Kollicoat^®^ Smartseal films in NaCl solution and the slower drug release from NaCl-containing pellets in stomach-mimicking pH conditions by the salting-out effect [40,41,42].

The fluid bed coating method provided here is well-accepted and widespread in the pharmaceutical industry. Laboratory- and industrial-scale fluid bed coaters are present in many manufacturing facilities worldwide. The proposed manufacturing process does not require organic solvents, which could be a limitation for some drug product manufacturers. The proposed cores and other excipients are commercially available. Thus, the proposed technology does not have barriers to industrialisation and can be easily adopted by manufacturers to produce personalised medical products.

Nevertheless, at the current stage, the proposed formulations are not ready to be proposed for clinical implementation. Despite achieving desirable taste-masking properties, the drug release profiles shown here do not meet immediate release [11]. Thus, considering favourable industrialisation possibilities, further study on formulation and drug release optimisation is reasonable.

## 4. Conclusions

Three types of cores (PharSQ^®^ Spheres M, CELPHERE™ CP-507, and NaCl) with comparable particle sizes were successfully warfarin-loaded and taste-masking-coated. The cores were coated with Kollicoat^®^ Smartseal 30D at a level of approximately 2 mg/cm^2^ and none of the pellets demonstrated drug release in saliva-simulated pH, confirming their efficient taste-masking. At a comparable coating level, in a stomach-simulated environment, PharSQ^®^ Spheres M and CELPHERE™ CP-507 showed a faster drug release in comparison to NaCl cores (91 and 92 versus 75% per 60 min, respectively). The observation of single-pellet behaviour in 0.1 N HCl and isolated film behaviour in 0.1 N HCl with and without NaCl was used to confirm the salting-out effect of NaCl on the Kollicoat^®^ Smartseal and justify the slower drug release from NaCl-containing pellets. The resulting pellets allow for the personalisation of the dose of warfarin (between 2 and 10 mg per day) and improve compliance due to taste masking and facilitated swallowing. Nevertheless, the drug release did not meet the immediate release requirements for any of the pellets.

## Figures and Tables

**Table 1:** The drug-layering composition.

Ingredients	per 100 g	Solids, g	Solids, wt.%
Warfarin sodium clathrate (raw substance)	28.7	–	–
Equivalent of warfarin sodium	26.3	26.3	95.3
Methocel™ E5	1.2	1.2	4.3
Glycerin (99.9%)	0.1	0.1	0.4
Water	70.0	–	–

**Table 2:** The composition of taste-masking coating.

Ingredients	per 100 g	Solids, g	Solids, wt.%
Kollicoat^®^ Smartseal 30D	59.0	–	–
Equivalent of Kollicoat^®^ Smartseal (solids)	17.7	17.7	88.5
Dibutyl sebacate	2.3	2.30	11.5
Added purified water	38.7	–	–

**Table 3:** The values of practical WGs achieved upon drug-layering and taste-masking coating.

	NaCl	CELPHERE™ CP-507	PharSQ^®^ Spheres M
**Drug layering (WG, wt.%)**	28.5	27.5	27.5
**Taste-masking coating (WG, wt.%)**	14.2	15.2	14.0

**Table 4:** Measured and calculated properties of pellets.

		NaCl			CELPHERE™ CP-507			PharSQ^®^ Spheres M		
		Cores	Cores—DL	Cores—DL-TM	Cores	Cores—DL	Cores—DL-TM	Cores	Cores—DL	Cores-DL-TM
**Size** ^A^, µm	Av.	521	549	668	615	698	738	532	570	590
(*n* = 150)	S.D.	35	51	49	49	61	51	17	19	21
	min	457	458	567	492	579	620	485	520	527
	max	646	749	813	747	912	887	609	650	650
**Volume** ^B^, mm^3^	Av.	0.143	0.170	0.303	0.124	0.182	0.213	0.079	0.097	0.108
	S.D.	0.031	0.051	0.068	0.030	0.051	0.045	0.008	0.010	0.011
	min	0.095	0.096	0.182	0.062	0.101	0.124	0.060	0.074	0.077
	max	0.270	0.419	0.537	0.218	0.397	0.365	0.118	0.143	0.143
**150 pellets** ^B^, mm^3^	–	21.5	25.5	45.4	18.6	27.3	32.0	11.9	14.6	16.2
**150 pellets** ^A^, g	Av.	0.051	0.057	0.063	0.023	0.034	0.043	0.019	0.025	0.030
(*n* = 3)	S.D.	0.001	0.001	0.001	0.000	0.001	0.002	0.000	0.001	0.000
**SSA** ^B^, cm^2^/g	–	48.4	47.7	63.8	77.6	67.3	60.4	70.4	62.0	54.0
**Apparent density** ^B^, g/mL	–	2.36	2.25	1.39	1.24	1.26	1.33	1.60	1.69	1.88

A—measured; B—calculated; SSA—specific surface area.

**Table 5:** Particle size distribution of pellets’ cores.

	NaCl	CELPHERE™ CP-507	PharSQ^®^ Spheres M
D_10%_, µm	481.7	500.2	439.8
D_50%_, µm	601.5	585.7	500.3
D_90%_, µm	756.5	686.8	555.9
Span	0.46	0.32	0.23

**Table 6:** The composition of pellets.

Composition, wt.%	NaCl	CELPHERE™ CP-507	PharSQ^®^ Spheres M
**Cores**	**67.8**	**67.2**	**67.9**
**Drug layer**	**19.3**	**19.2**	**19.4**
***Warfarin sodium***	*18.4*	*18.3*	*18.4*
*Methocel™ E5*	*0.8*	*0.8*	*0.8*
*Glycerine*	*0.1*	*0.1*	*0.1*
**Taste-masking layer**	**12.4**	**13.1**	**12.2**
*Kollicoat^®^ Smartseal (solids)*	*11.0*	*11.6*	*10.8*
*Dibutyl sebacate*	*1.4*	*1.5*	*1.4*
Syloid^®^ 244FP	**0.5**	**0.5**	**0.5**
∑	**100.0**	**100.0**	**100.0**

The layers are indicated in bold, and the layer components are in italics.

**Figure A1:**
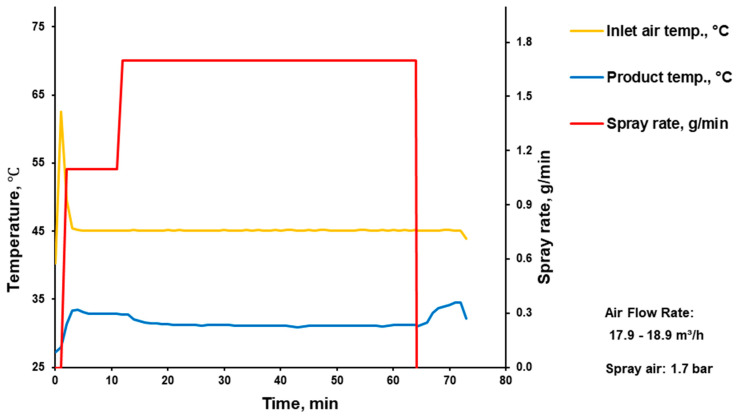
Drug loading of PharSQ^®^ Spheres M cores.

**Figure A2:**
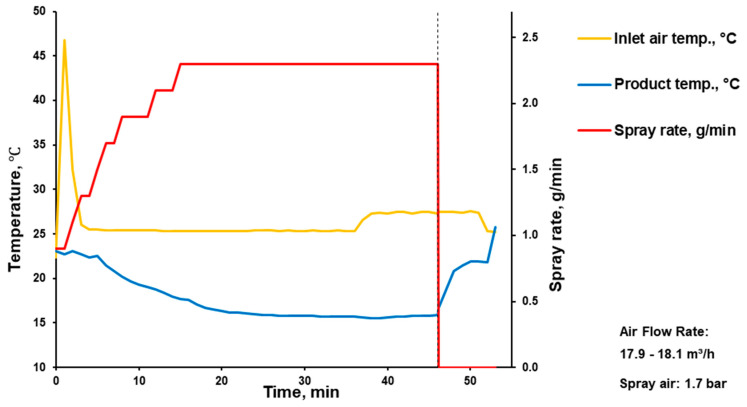
Taste-masking coating of drug-loaded PharSQ^®^ Spheres M cores.

**Figure A3:**
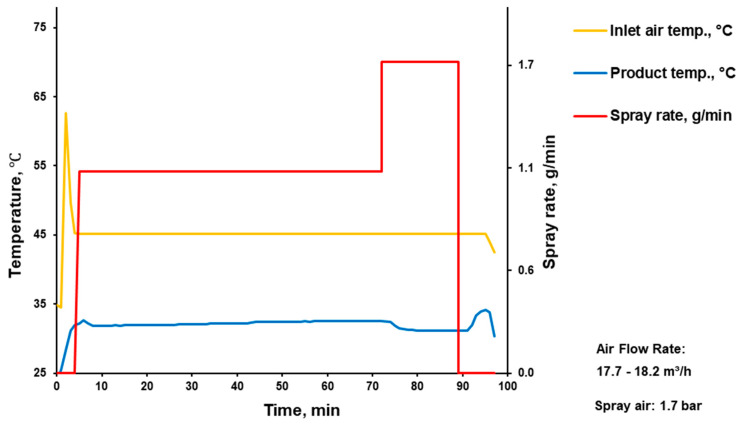
Drug loading of CELPHERE™ CP-507 cores.

**Figure A4:**
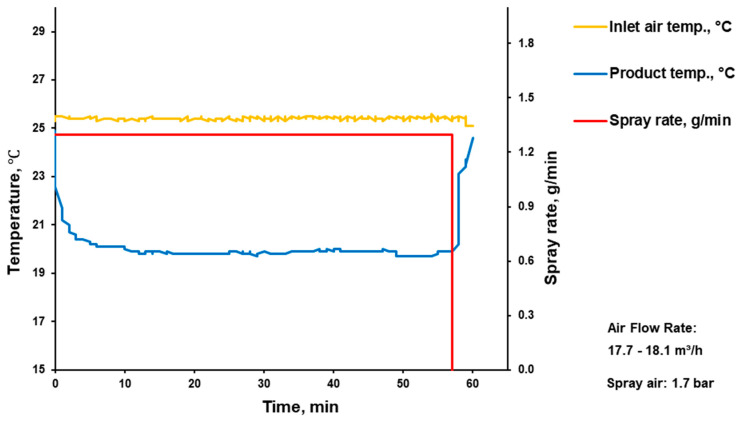
Taste-masking coating of drug-loaded CELPHERE™ CP-507 cores.

**Figure A5:**
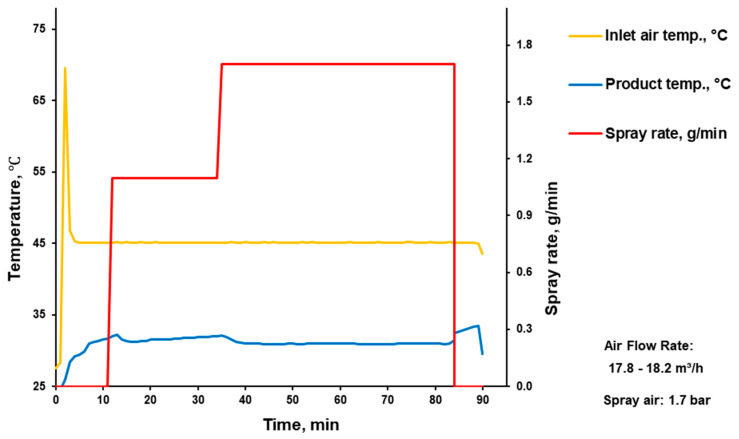
Drug loading of NaCl cores.

**Figure A6:**
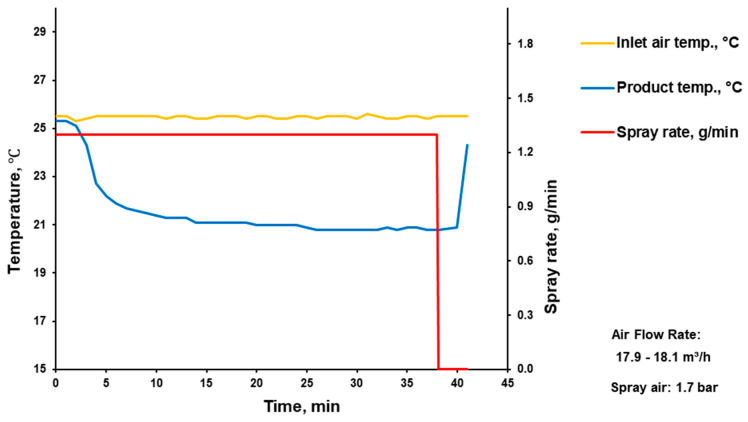
Taste-masking coating of drug-loaded NaCl cores.

## Data Availability

The data presented in this study are available on request from the corresponding author.
